# Post-transcriptional control of HIV-1 Gag expression

**DOI:** 10.1186/1742-4690-10-S1-P87

**Published:** 2013-09-19

**Authors:** Prabhjeet Phalora, Charlotte Mahiet, Michael Malim, Chad Swanson

**Affiliations:** 1 Department of Infectious Diseases, King’s College London School of Medicine, London SE1 9RT, UK

## Background

The HIV-1 genomic RNA (gRNA) has several roles in the viral life cycle. In the nucleus, the gRNA is the pre-mRNA that is alternatively and incompletely spliced into mRNAs encoding Tat, Rev, Nef, Vif, Vpr, Vpu and Env. However, approximately 50% of the gRNA remains unspliced and is exported from the nucleus by the viral protein Rev using the CRM1 nuclear export pathway. In the cytoplasm, the gRNA serves as the mRNA for Gag and Gag-Pol and is also packaged into virions. As the gRNA is an unspliced, intron-containing mRNA with a long, highly structured 5’ UTR and rare codon usage in the open reading frames, how it interacts with cellular RNA metabolism and translation machinery to promote abundant Gag expression and infectious virion production remains unclear.

## Materials and methods

To identify and characterise cellular RNA binding proteins that regulate HIV-1 Gag expression, we use a *Gag-Pol* mini-gene that contain the 5’ UTR, Gag-Pol open reading frames and Rev-response element (RRE). HIV-1 Gag expression is determined by quantitative western blotting. gRNA abundance and splicing are analysed by northern blotting. The Gag translation rate is quantified by ^35^S cysteine-methionine pulse labelling.

## Results

To identify cellular factors that regulate gRNA splicing, abundance and/or translation efficiency, we took advantage of the fact that mouse cells are non-permissive for HIV-1 Gag expression. We screened candidate cDNAs encoding human proteins that interact with the gRNA for their ability to increase Gag expression and virion production in mouse cells. From this screen, we have identified proteins from several gene families whose overexpression stimulates Gag expression. Ectopic expression of human CRM1, but not mouse CRM1, rescues Rev-dependent nuclear export of HIV-1 gRNA. However, this does not completely rescue Gag expression in mouse cells. Interestingly, overexpression of specific members of the SR and STAR families of RNA binding proteins stimulate Gag expression and are additive with human CRM1 expression. The active SR proteins regulate Gag expression by inhibiting gRNA splicing and increasing Gag translation efficiency, indicating that they couple nuclear and cytoplasmic gRNA regulation.

## Conclusions

Overexpression of specific members of the SR and STAR RNA binding protein families rescue the block in Gag expression in mouse cells that remains even when gRNA nuclear export is rescued by human CRM1 expression. We are currently characterising the molecular mechanisms by which these proteins stimulate Gag expression and are using a combination of RNAi and potential dominant interfering mutants of the SR and STAR proteins to analyse how they control Gag expression in human cells.

